# Associations of polyunsaturated fatty acids with cardiovascular disease and mortality: a study of NHANES database in 2003–2018

**DOI:** 10.1186/s12902-023-01412-4

**Published:** 2023-08-29

**Authors:** Na Zhong, Pengyu Han, Yulin Wang, Chaoyang Zheng

**Affiliations:** 1 Department of Cardiology, Shunde Hospital of Guangzhou University of Chinese Medicine, Foshan, Guangdong 528311 People’s Republic of China; 2 Department of Cardiovascularology, The Second Clinical Medical College of Guangzhou, University of Traditional Chinese Medicine, No. 12, Jinshada Road, Daliang Town, Shunde District, Guangzhou, Guangdong 510006 People’s Republic of China

**Keywords:** Diet, Retrospective cohort, Mortality risk, Population-based study

## Abstract

**Background:**

This study was to explore the association between dietary polyunsaturated fatty acids (PUFAs) consumption and cardiovascular diseases (CVDs), all-cause mortality, and CVD-specific mortality.

**Methods:**

This retrospective cohort study extracted demographic and clinical data of 38,838 adult participants from the National Health and Nutrition Examination Survey (NHANES) database in 2003–2018. We explored the association between octadecadienoic acid (ODA), octadecatrienoic acid (ALA), octadecatetraenoic acid (ODTA), eicosatetraenoic acid (AA), eicosapentaenoic acid (EPA), docosapentaenoic acid (DPA), and docosahexaenoic acid (DHA) and different CVDs using weighted univariate and multivariate logistic regression analyses with odds ratio (OR) and 95% confidence interval (CI). The PUFAs were divided into four levels according to the quartiles (≤ Q1, Q1 to Q2, Q1 to Q2, > Q3). Weighted univariate and multivariate COX regression analyses with hazard ratio (HR) and 95% CI were used for exploring the association between PUFAs and all-cause mortality, CVD-specific mortality and other cause-specific mortality.

**Results:**

During the follow-up, a total of 4,908 (9.12%) eligible participants died. The results showed that after adjusting for covariates, ODTA intake was related to low odds of coronary heart disease (CHD) [OR = 0.75, 95%CI: (0.64–0.88)]. Q1-Q2 quartile of ALA [OR = 0.81, 95%CI: (0.66–0.99)] and Q2-Q3 quartile of DPA [OR = 0.78, 95%CI: (0.62–0.99)] intakes were linked to low odds of heart attack, and > Q3 quartile of ODA intake was associated with low odds of congestive heart failure (CHF) [OR = 0.66, 95%CI: (0.49–0.90)] and stroke [OR = 0.65, 95%CI: (0.47–0.90)]. Q2-Q3 quartile of DPA intake was linked to low odds of angina [OR = 0.76, 95%CI: (0.58–0.99)]. Higher ALA intake was associated with a lower risk of all-cause mortality [Q2-Q3: HR = 0.86, 95%CI: (0.74–0.99); > Q3: HR = 0.76, 95%CI: (0.63–0.91)]. Additionally, Q2-Q3 quartile of ALA, Q1-Q2 quartile of AA and DPA intakes were respectively related to a low risk of CVD-specific mortality, while that > Q3 quartile of ALA related to that of mortality by other causes.

**Conclusion:**

Our study found that PUFAs were associated with different CVDs, and higher ALA intake was related to lower risk of all-cause mortality. Ensuring adequate intake of PUFAs was beneficial to the health and may decrease the risk of mortality.

**Supplementary Information:**

The online version contains supplementary material available at 10.1186/s12902-023-01412-4.

## Introduction

Following a healthy diet can delay the onset of cardiovascular disease (CVD) and some chronic diseases and prolong life [1–3]. Polyunsaturated fatty acids (PUFAs), with the food sources including fish, vegetable oils, poultry meat and eggs and so on [4–6], have been widely reported to play important roles in regulating a variety of physiological processes, such as inflammation, glucose regulation, lipid metabolism, and oxidative stress [7, 8]. PUFAs are involved in the progression of neurological, cardiovascular system and cancer-related diseases by regulating lipid metabolism [9].

High saturated fatty acids (SFAs) intake has been reported to be linked to triglycerides to HDL-cholesterol ratio (TG/HDL-cholesterol) levels, and is an independent risk factor of CVD [10]. Eating large amounts of SFAs may result in increased cholesterol synthesis, cause disturbances of lipid metabolism in liver, and is further associated with atherosclerosis [11]. Therefore, growing recommendations suggested partial replacement of saturated fats in the diet with PUFAs to reduce the risk of certain metabolic disorders or cardiovascular diseases (CVDs), which are leading causes of mortality [12].

PUFAs such as octadecatrienoic acid (ALA), eicosapentaenoic acid (EPA), and docosahexaenoic acid (DHA), linoleic acid (LA), and arachidonic acid (AA) are important for human health [13]. According to a meta-analysis of 44 prospective cohort studies, high intake of linoleic acid (LA) was associated with a modest reduction in the risk of mortality from CVD and cancer [14]. ALA is metabolized to EPA and DHA, both of which are anti-inflammatory characteristics [15]. Naghshi et al. found that dietary intake of ALA was associated with a low risk of mortality from CVD and coronary heart disease (CHD) [15]. Djuricic et al. showed that EPA and DHA are not only related to lower incidence of chronic diseases characterized by elevated inflammation, including CVDs, but also can regulate homeostasis of platelet and lower the risk of thrombosis [16]. Although many studies have investigated associations of PUFAs with cardiovascular outcomes, relatively few have examined other endpoints, particularly all-cause mortality [17].

To our knowledge, studies on the association between dietary PUFAs consumption and all-cause mortality were still lacking [18]. Herein, this study aims to explore the relationship of PUFAs including octadecadienoic acid (ODA), ALA, octadecatetraenoic acid (ODTA), eicosatetraenoic acid (AA), EPA, docosapentaenoic acid (DPA), and DHA, and CVDs and the risk of all-cause mortality. And we hope to provide some dietary reference for reducing risk of CVDs and further reducing mortality.

## Methods

### Study design and participants

Publicly available data from the National Health and Nutrition Examination Survey (NHANES) database in 2003–2018 were used in this retrospective cohort study. The NHANES database uses a stratified multistage probability sampling approach to select a representative sample of the civilian uninstitutionalized U.S. population for the purpose of assessing the health and nutritional status of the U.S. population [19]. The NHANES data files can be accessed at the following links (http://www.cdc.gov/nhanes (accessed on 5 January 2022)).

A total of 47,763 adults were initially included. Then, those who with incomplete data of CVDs, unreliable 24-h dietary review data [20], and extreme total energy intake [20] were excluded. Finally, 38,838 of them were eligible. Participants with extreme total energy intake was defined as less than 500 or more than 5000 kcal/day for women and less than 500 or more than 8000 kcal/day for men. The National Center for Health Statistics (NCHS) Ethics Review Committee (ERC) granted ethics approval of NHANES database, and all individuals provided written informed consent before participating in the survey. The requirement of ethical approval for this study was waived by the Institutional Review Board (IRB) of The Second Clinical Medical College of Guangzhou University of Traditional Chinese Medicine, because the data was accessed from NHANES (a publicly available database). All methods were carried out in accordance with relevant guidelines and regulations (declaration of Helsinki).

### Outcomes

The primary outcome was all-cause mortality and secondary outcomes were CVD-specific mortality and other cause-specific mortality. Mortality information was obtained from the linked data provided by the Centers for Disease Control and Prevention (CDC) (https://www.cdc.gov/nchs/data-linkage/mortality-public.htm (accessed on 11 January 2022)). The definition for all-cause mortality was based on the International Classification of Diseases, 10th revision (ICD-10), and was assessed through the National Death Index (NCHS). CVD-specific mortality was assessed using ICD I00-I09, I11, I13, I20-I51, and I60-I69 [21]. The follow-up ended until the last known date alive or censored through 31 December 2019.

### Dietary polyunsaturated fatty acids measurement

Dietary information was measured using two 24-h dietary recall surveys in NHANES [22]. The first 24-h recall interview was conducted in person in the mobile exam centers (MECs) by trained interviewers, and the second interview was performed by telephone or mail three to ten days later. We used the records of first 24-h recall in this study. The main exposures were dietary PUFAs intake including ODA, ALA, ODTA, AA, EPA, DPA, and DHA. We divided the PUFAs into three levels according to the quartiles (≤ Q1, Q1 to Q2, Q1 to Q2, > Q3), and the specific cut-offs for each PUFA were showed in Table S1.

### Covariates

Covariates included the following demographic characteristics: age, gender, race (Mexican American, other Hispanic, non-Hispanic White, non-Hispanic Black, or other race), educational level [less than 9th grade, 9-11th grade (includes 12th grade with no diploma), high school graduate/general educational development (GED) or equivalent, or more than high school], marital status (married or living with partner, widowed/ divorced/separated, or never married), poverty income ratio (PIR), body mass index (BMI, kg/m^2^), physical activity (low level or high level), total energy intake [by two 24-h dietary recall interviews and two 24-h dietary supplement recall interviews], history of hypertension (the participants have been told by the doctor have hypertension), history of diabetes mellitus (DM, the participants have been told by the doctor have diabetes), smoking (smoking at least 100 cigarettes in life), and drinking (having at least 12 alcohol drinks of any type in any given year). Physical activity was converted to metabolic equivalent (MET), which was calculated according to the physical activity questionnaire (PAQ) in NHANES. Energy expenditure (MET·min) = recommended MET × exercise time of corresponding activity (min).

### Statistical analysis

Normal distribution data were described using mean ± standard error (mean ± SE) and independent-samples analysis of variance (ANOVA) for group comparation. Enumeration data were expressed as count and constituent ratio [N (%)] and chi-square test for the comparison. We used a set of weights “WTDRD1” because we used dietary recall data of the first 24-h for analyses. The first 24-h weights were constructed by taking the MEC sample weights (WTMEC2YR) and further adjusting for (a) the additional non-response and (b) the differential allocation by day of the week for the dietary intake data collection (https://wwwn.cdc.gov/Nchs/Nhanes/2007-2008/DR1IFF_D.htm#WTDRD1).

Univariate and multivariate logistic regression analyses with odds ratio (OR) and 95% confidence interval (CI) were used to explore the association between PUFAs and CVDs including CHD, heart attack, congestive heart failure (CHF), stroke and angina. The relationships of PUFAs and all-cause mortality, CVD-specific mortality, and other cause-specific mortality were analyzed respectively using univariate and multivariate Cox regression with hazard ratio (HR) and 95% CI. Model 1 was the crude model. Model 2 adjusted for age, gender, race, education level, marital status, PIR, BMI, physical activity, hypertension, DM, smoking, drinking, and total energy intake. Model 3 adjusted for different PUFAs (excluding the one for analysis) in addition to covariates in Model 2. The significance level was set at α = 0.05, and all analyses were performed using R v. 4.1.2 (R Foundation for Statistical Computing, Vienna, Austria).

## Results

### Characteristics of the study population

**Fig. 1:**
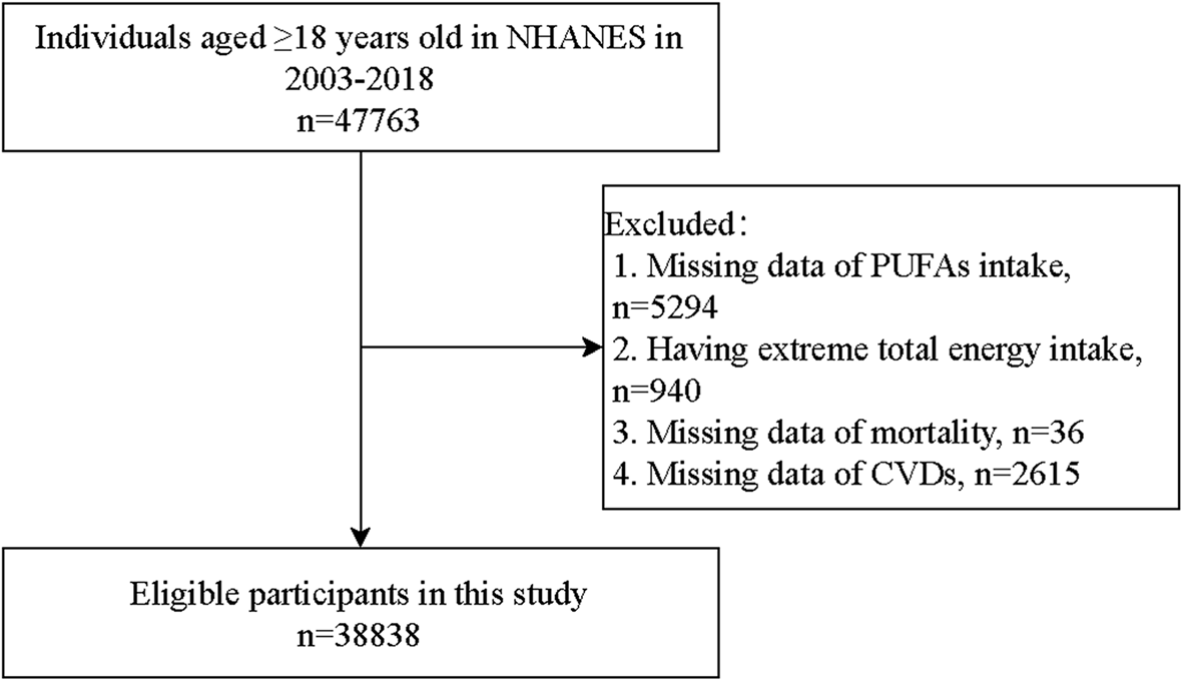
Flow chart of study population screening

We initially included 47,763 adults from NHANES in 2003–2018. Among them, the individuals without complete data of PUFAs intake (*n* = 5294) and CVDs (2615), missing death information (*n* = 36), and with extreme total energy intake (*n* = 940) were excluded. Finally, a total of 38,838 participants were eligible (Fig. 1).

The characteristics of study population was showed in Table 1. During average 100 months follow-up, 4,908 (9.12%) of them died for all-cause. The average age was 47 years old, and 52.39% of them were women and 47.62% were men. There were 10,600 participants had a PUFAs intake level ≤ Q1, 9,887 in the PUFAs Q1-Q2 group, 9,285 in the PUFAs Q2-Q3 group, and 9,066 in the PUFAs > Q3 group. Among the four PUFAs intake level groups, age, gender, race, education level, marital status, PIR, BMI, physical activity, total energy intake, hypertension, DM, smoking, and drinking were significantly different (all *P* < 0.001).

**Table 1:** Characteristics of participants in different quartiles of PUFAs group

Variables	Total (*n* = 38,838)	PUFAs intake levels				Statistics	*P*
		≤ Q1 (*n* = 10,600)	Q1-Q2 (*n* = 9887)	Q2-Q3 (*n* = 9285)	> Q3 (*n* = 9066)		
Age, years, Mean (S.E)	47.44 (0.22)	49.15 (0.31)	48.26 (0.28)	46.83 (0.33)	45.52 (0.29)	F = 48.36	< 0.001
Gender, n (%)						χ^2^ = 688.024	< 0.001
Male	18,656 (47.62)	4073 (35.26)	4453 (43.64)	4699 (50.47)	5431 (61.09)		
Female	20,182 (52.39)	6527 (64.74)	5434 (56.36)	4586 (49.53)	3635 (38.92)		
Race, n (%)						χ^2^ = 85.284	< 0.001
Mexican American	6284 (8.48)	1724 (8.11)	1681 (9.10)	1513 (8.66)	1366 (8.04)		
Other Hispanic	3399 (5.13)	1126 (6.22)	888 (5.17)	767 (4.96)	618 (4.17)		
Non-Hispanic White	17,108 (67.81)	4516 (65.72)	4348 (67.87)	4171 (68.56)	4073 (69.09)		
Non-Hispanic Black	8248 (11.24)	2121 (11.51)	1992 (10.51)	1940 (10.52)	2195 (12.44)		
Other Race—Including Multi-Racial	3799 (7.34)	1113 (8.44)	978 (7.36)	894 (7.30)	814 (6.26)		
Education level, n (%)						χ^2^ = 287.487	< 0.001
Less than 9th grade	4208 (5.37)	1693 (8.29)	1106 (5.61)	812 (4.35)	597 (3.24)		
9-11th grade (Includes 12th grade with no diploma)	5523 (10.57)	1722 (12.85)	1434 (11.09)	1219 (9.23)	1148 (9.12)		
High school graduate/ GED or Equivalent	9032 (23.83)	2424 (24.83)	2339 (24.20)	2203 (23.14)	2066 (23.14)		
More than high school	20,075 (60.23)	4761 (54.04)	5008 (59.09)	5051 (63.28)	5255 (64.50)		
Marital status, n (%)						χ^2^ = 104.205	< 0.001
Married or living with partner	23,260 (62.48)	5949 (58.14)	5924 (62.53)	5748 (63.99)	5639 (65.27)		
Widowed/Divorced/Separated	8697 (18.95)	2895 (23.44)	2261 (19.77)	1886 (17.08)	1655 (15.51)		
Never married	6881 (18.57)	1756 (18.43)	1702 (17.71)	1651 (18.93)	1772 (19.23)		
PIR, Mean (S.E)	2.97 (0.03)	2.70 (0.04)	2.96 (0.03)	3.07 (0.04)	3.13 (0.04)	F = 45.30	< 0.001
BMI, kg/m^2^, Mean (S.E)	28.94 (0.08)	28.79 (0.11)	28.76 (0.11)	28.92 (0.11)	29.28 (0.12)	F = 6.14	< 0.001
Physical activity, MET·min, n (%)						χ^2^ = 144.409	< 0.001
Low level	19,618 (43.96)	6012 (49.92)	5150 (46.77)	4394 (40.12)	4062 (39.04)		
High level	19,220 (56.04)	4588 (50.08)	4737 (53.23)	4891 (59.88)	5004 (60.96)		
Total energy intake, kcal, Mean (S.E)	2117.97 (7.05)	1387.19 (7.39)	1877.24 (8.38)	2268.68 (10.59)	2938.63 (12.62)	F = 4239.45	< 0.001
Hypertension, n (%)						χ^2^ = 45.625	< 0.001
No	19,351 (55.27)	4806 (51.66)	4827 (54.74)	4823 (57.45)	4895 (57.22)		
Yes	19,487 (44.73)	5794 (48.34)	5060 (45.26)	4462 (42.56)	4171 (42.78)		
DM, n (%)						χ^2^ = 40.146	< 0.001
No	27,366 (74.86)	7126 (72.03)	6846 (73.96)	6695 (76.47)	6699 (76.97)		
Yes	11,472 (25.14)	3474 (27.97)	3041 (26.04)	2590 (23.53)	2367 (23.03)		
Smoking, n (%)						χ^2^ = 52.294	< 0.001
No	21,280 (54.25)	5905 (53.95)	5462 (54.12)	5121 (55.69)	4792 (53.25)		
Yes	8883 (23.45)	2432 (25.79)	2242 (24.10)	2034 (20.63)	2175 (23.26)		
Quitting	8654 (22.27)	2255 (20.23)	2179 (21.77)	2125 (23.62)	2095 (23.46)		
Unknown	21 (0.04)	8 (0.04)	4 (0.02)	5 (0.06)	4 (0.03)		
Drinking, n (%)						χ^2^ = 140.868	< 0.001
No	5115 (10.36)	1884 (14.09)	1310 (10.53)	1057 (9.29)	864 (7.53)		
Yes	5743 (15.60)	1448 (13.46)	1414 (15.27)	1401 (16.80)	1480 (16.87)		
Quitting	2958 (6.76)	834 (7.35)	801 (7.10)	684 (6.40)	639 (6.17)		
Unknown	25,022 (67.29)	6434 (65.11)	6362 (67.10)	6143 (67.51)	6083 (69.44)		
All-cause mortality, n (%)						χ^2^ = 254.579	< 0.001
No	33,930 (90.88)	8810 (87.25)	8521 (89.83)	8269 (92.23)	8330 (94.21)		
Yes	4908 (9.12)	1790 (12.75)	1366 (10.17)	1016 (7.77)	736 (5.79)		
Follow-up time, months, Mean (S.E)	100.27 (1.13)	104.19 (1.28)	102.06 (1.32)	98.92 (1.37)	95.91 (1.50)	F = 14.54	< 0.001

*PUFA* Polyunsaturated fatty acid, *Q1* 1st quartile, *Q2* 2nd quartile, *Q3* 3rd quartile, *SE* Standard error, *PIR* Poverty income ratio, *BMI* Body mass index, *DM* Diabetes mellitus

*F* Analysis of variance, χ^2^: chi-squared test

### Association between PUFAs and the risk of CVDs

We first explored the relationships between PUFAs intake and the risk of CVDs (Table 2). After adjusting for the covariates, we found that ODTA intake was related to low odds of CHD [OR = 0.75, 95%CI: (0.64–0.88), *P* < 0.001], Q1-Q2 quartile of ALA [OR = 0.81, 95%CI: (0.66–0.99), *P* = 0.043] and Q2-Q3 quartile of DPA [OR = 0.78, 95%CI: (0.62–0.99), *P* = 0.037] intakes were linked to low odds of heart attack, > Q3 quartile of ODA intake was associated with low odds of CHF [OR = 0.66, 95%CI: (0.49–0.90), *P* = 0.009] and stroke [OR = 0.65, 95%CI: (0.47–0.90), *P* = 0.009], and Q2-Q3 quartile of DPA intake was linked to low odds of angina [OR = 0.76, 95%CI: (0.58–0.99), *P* = 0.043].

**Table 2:** Association of different PUFAs concentrations with the risk of CHD, heart attack, CHF, stroke, and angina

PUFAs level	CHD		Heart attack		CHF		Stroke		Angina	
	OR (95% CI)	*P*	OR (95% CI)	*P*	OR (95% CI)	*P*	OR (95% CI)	*P*	OR (95% CI)	*P*
**ODA**										
≤ Q1	Ref		Ref		Ref		Ref		Ref	
Q1-Q2	1.17 (0.94–1.46)	0.154	1.03 (0.85–1.24)	0.785	0.86 (0.71–1.04)	0.125	0.91 (0.75–1.10)	0.330	0.87 (0.65–1.15)	0.327
Q2-Q3	1.05 (0.80–1.38)	0.721	1.03 (0.77–1.37)	0.831	0.68 (0.54–0.86)	0.001	0.77 (0.59–1.01)	0.063	1.02 (0.76–1.37)	0.884
> Q3	1.15 (0.79–1.66)	0.463	0.97 (0.67–1.41)	0.888	0.66 (0.49–0.90)	0.009	0.65 (0.47–0.90)	0.009	1.02 (0.69–1.50)	0.915
**ALA**										
≤ Q1	Ref		Ref		Ref		Ref		Ref	
Q1-Q2	0.86 (0.69–1.07)	0.176	0.81 (0.66–0.99)	0.043	1.02 (0.80–1.29)	0.893	0.96 (0.78–1.19)	0.717	0.96 (0.75–1.24)	0.758
Q2-Q3	0.81 (0.63–1.05)	0.117	0.73 (0.53–1.01)	0.058	1.07 (0.82–1.39)	0.624	0.90 (0.69–1.16)	0.402	0.86 (0.63–1.17)	0.328
> Q3	0.74 (0.53–1.04)	0.079	0.72 (0.50–1.04)	0.079	1.39 (0.99–1.94)	0.057	1.03 (0.75–1.42)	0.836	0.77 (0.52–1.13)	0.178
**ODTA**										
No	Ref		Ref		Ref		Ref		Ref	
Yes	0.75 (0.64–0.88)	< 0.001	0.90 (0.76–1.06)	0.198	0.91 (0.74–1.12)	0.368	0.87 (0.71–1.08)	0.202	0.82 (0.66–1.02)	0.069
**AA**										
≤ Q1	Ref		Ref		Ref		Ref		Ref	
Q1-Q2	0.98 (0.81–1.18)	0.814	0.95 (0.77–1.17)	0.642	0.86 (0.69–1.07)	0.179	1.06 (0.87–1.29)	0.585	0.86 (0.67–1.10)	0.219
Q2-Q3	1.00 (0.79–1.27)	0.989	1.05 (0.79–1.39)	0.758	1.10 (0.86–1.40)	0.442	1.22 (0.97–1.54)	0.094	0.96 (0.71–1.30)	0.811
> Q3	0.90 (0.67–1.20)	0.463	1.01 (0.77–1.33)	0.942	1.07 (0.80–1.42)	0.653	1.04 (0.76–1.43)	0.817	0.94 (0.66–1.35)	0.741
**EPA**										
≤ Q1	Ref		Ref		Ref		Ref		Ref	
Q1-Q2	1.06 (0.89–1.27)	0.491	1.03 (0.84–1.26)	0.767	0.94 (0.76–1.14)	0.511	0.85 (0.68–1.06)	0.141	1.03 (0.82–1.29)	0.794
Q2-Q3	0.90 (0.72–1.13)	0.353	1.02 (0.83–1.27)	0.828	0.80 (0.62–1.04)	0.096	1.09 (0.85–1.39)	0.488	1.10 (0.80–1.52)	0.562
> Q3	1.30 (0.94–1.79)	0.114	1.12 (0.83–1.51)	0.468	0.93 (0.69–1.26)	0.648	0.92 (0.69–1.24)	0.594	1.40 (0.95–2.07)	0.092
**DPA**										
≤ Q1	Ref		Ref		Ref		Ref		Ref	
Q1-Q2	1.07 (0.85–1.33)	0.576	0.83 (0.68–1.02)	0.070	0.95 (0.75–1.20)	0.658	0.81 (0.64–1.03)	0.087	0.80 (0.60–1.07)	0.132
Q2-Q3	0.99 (0.81–1.22)	0.959	0.78 (0.62–0.99)	0.037	0.86 (0.66–1.13)	0.282	0.78 (0.59–1.03)	0.075	0.76 (0.58–0.99)	0.043
> Q3	1.12 (0.82–1.53)	0.458	0.84 (0.62–1.13)	0.248	0.99 (0.71–1.38)	0.956	0.94 (0.69–1.29)	0.699	0.85 (0.59–1.22)	0.368
**DHA**										
≤ Q1	Ref		Ref		Ref		Ref		Ref	
Q1-Q2	0.89 (0.70–1.13)	0.334	0.91 (0.71–1.17)	0.482	1.10 (0.89–1.36)	0.388	1.04 (0.84–1.29)	0.727	0.90 (0.67–1.21)	0.492
Q2-Q3	0.95 (0.73–1.23)	0.687	1.05 (0.80–1.38)	0.722	1.14 (0.89–1.46)	0.292	1.05 (0.81–1.36)	0.708	0.98 (0.71–1.35)	0.904
> Q3	0.73 (0.51–1.04)	0.083	1.00 (0.70–1.45)	0.980	0.85 (0.61–1.19)	0.336	1.03 (0.75–1.40)	0.870	0.80 (0.55–1.16)	0.240

*PUFA* Polyunsaturated fatty acid, *CHD* Coronary heart disease, *CH*F Congestive heart failure, *OR* Odds ratio, *CI* Confidence interval, *ODA* Octadecadienoic acid, *Ref*: Reference, *ALA* Octadecatrienoic acid, *ODTA* Octadecatetraenoic acid, *AA* Eicosatetraenoic acid, *EPA* eicosapentaenoic acid, *DPA* Docosapentaenoic acid, *DHA*: Docosahexaenoic acid

Model 1 was the crude model;

Model 2 adjusted for age, gender, race, education level, marital status, PIR, BMI, physical activity, hypertension, DM, smoking, drinking, total energy intake;

Model 3 adjusted for covariates adjusted in Model 2 as well as different PUFAs (excluded the one for analysis)

### Association between PUFAs and all-cause mortality, CVD-specific mortality and other cause-specific mortality

**Fig. 2:**
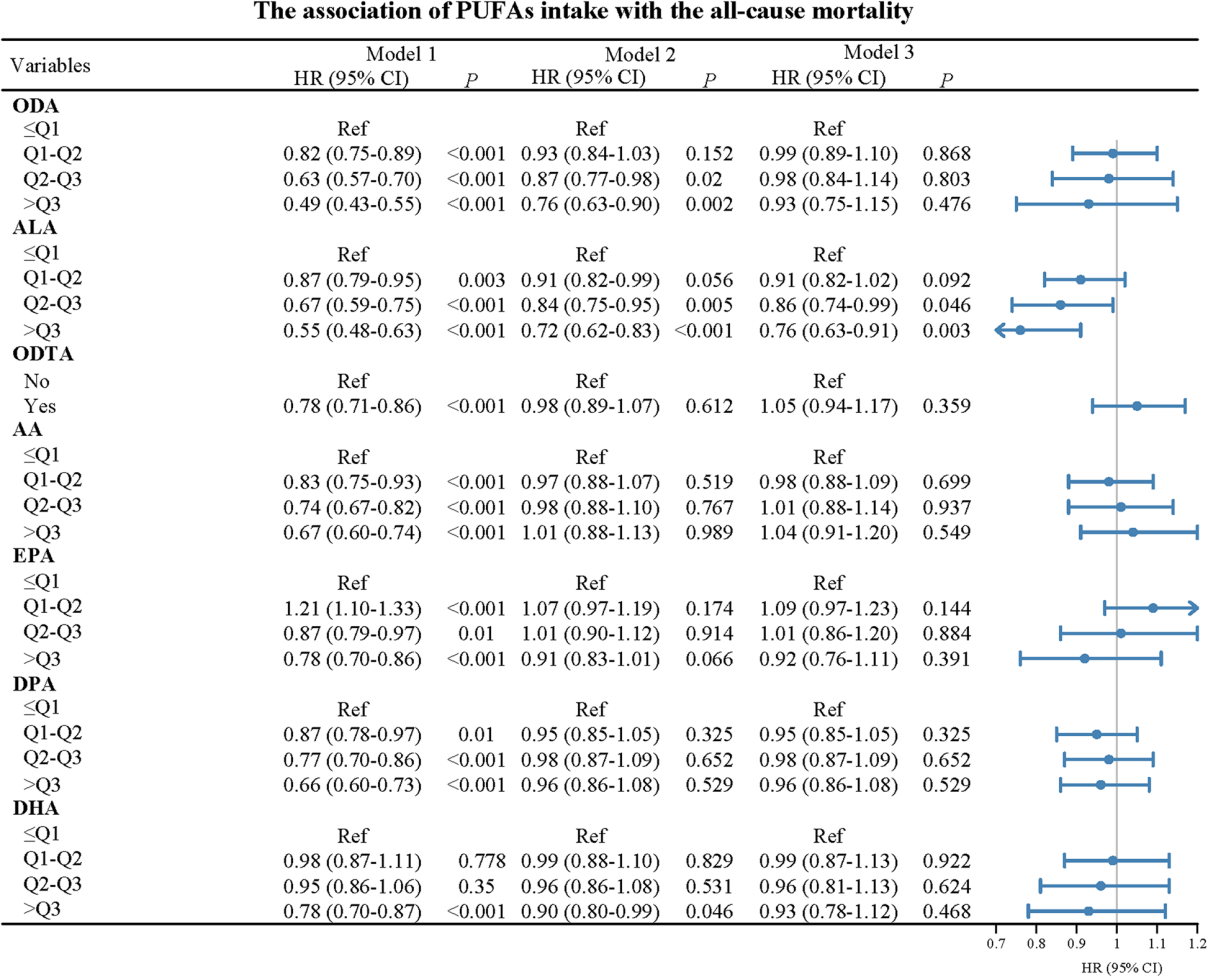
Association between dietary PUFAs intake and all-cause mortality

Figure 2 showed associations of PUFAs with all-cause mortality. After adjusting for covariates, in Model 3, only higher ALA intake was significantly related to lower risk of all-cause mortality [Q2-Q3: HR = 0.86, 95%CI: (0.74–0.99), *P* = 0.046; > Q3: HR = 0.76, 95%CI: (0.63–0.91), *P* = 0.003].

We further explored the relationship of PUFAs and CVD-specific mortality and other cause-specific mortality (Table 3). After adjusting for covariates, in Model 3, we found that Q2-Q3 quartile of ALA [OR = 0.74, 95%CI: (0.56–0.96), *P* = 0.026], Q1-Q2 quartile of AA [OR = 0.83, 95%CI: (0.71–0.97), *P* = 0.023] and DPA [OR = 0.72, 95%CI: (0.59–0.87), *P* < 0.001] intakes were respectively linked to a low risk of CVD-specific mortality, while that > Q3 quartile of ALA [OR = 0.70, 95%CI: (0.58–0.85), *P* < 0.001] related to that of mortality by other causes.

**Table 3:** Association between PUFAs and CVD-specific mortality and other cause-specific mortality

Variables	CVD-specific mortality						Other cause-specific mortality					
	Model 1		Model 2		Model 3		Model 1		Model 2		Model 3	
	HR (95% CI)	*P*	HR (95% CI)	*P*	HR (95% CI)	*P*	HR (95% CI)	*P*	HR (95% CI)	*P*	HR (95% CI)	*P*
**ODA**												
≤ Q1	Ref		Ref		Ref		Ref		Ref		Ref	
Q1-Q2	0.80 (0.68–0.93)	0.005	0.94 (0.80–1.11)	0.452	1.08 (0.90–1.31)	0.393	0.82 (0.73–0.93)	0.001	0.92 (0.81–1.04)	0.185	0.98 (0.85–1.13)	0.786
Q2-Q3	0.62 (0.52–0.74)	< 0.001	0.89 (0.73–1.09)	0.255	1.09 (0.85–1.41)	0.482	0.62 (0.55–0.71)	< 0.001	0.83 (0.72–0.95)	0.006	0.95 (0.79–1.13)	0.528
> Q3	0.42 (0.33–0.54)	< 0.001	0.73 (0.52–1.02)	0.065	0.90 (0.58–1.37)	0.609	0.51 (0.43–0.59)	< 0.001	0.73 (0.60–0.87)	< 0.001	0.94 (0.75–1.17)	0.549
**ALA**												
≤ Q1	Ref		Ref		Ref		Ref		Ref		Ref	
Q1-Q2	0.85 (0.72–0.99)	0.054	0.86 (0.72–1.02)	0.085	0.82 (0.67–1.01)	0.056	0.87 (0.77–0.97)	0.014	0.90 (0.80–1.01)	0.083	0.91 (0.80–1.04)	0.166
Q2-Q3	0.60 (0.48–0.74)	< 0.001	0.76 (0.61–0.94)	0.012	0.74 (0.56–0.96)	0.026	0.69 (0.60–0.79)	< 0.001	0.83 (0.72–0.96)	0.011	0.86 (0.72–1.02)	0.090
> Q3	0.53 (0.42–0.67)	< 0.001	0.71 (0.55–0.93)	0.012	0.77 (0.56–1.06)	0.104	0.54 (0.46–0.63)	< 0.001	0.66 (0.56–0.78)	< 0.001	0.70 (0.58–0.85)	< 0.001
**ODTA**												
No	Ref		Ref		Ref		Ref		Ref		Ref	
Yes	0.76 (0.65–0.90)	0.002	0.98 (0.84–1.13)	0.760	1.10 (0.94–1.28)	0.222	0.78 (0.71–0.87)	< 0.001	0.98 (0.87–1.10)	0.700	1.05 (0.92–1.19)	0.472
**AA**												
≤ Q1	Ref		Ref		Ref		Ref		Ref		Ref	
Q1-Q2	0.68 (0.58–0.81)	< 0.001	0.81 (0.69–0.94)	0.008	0.83 (0.71–0.97)	0.023	0.89 (0.79–1.01)	0.067	1.02 (0.91–1.15)	0.704	1.05 (0.91–1.20)	0.522
Q2-Q3	0.74 (0.62–0.89)	0.001	1.02 (0.85–1.22)	0.813	0.99 (0.82–1.22)	0.980	0.74 (0.65–0.83)	< 0.001	0.96 (0.84–1.10)	0.583	1.01 (0.86–1.18)	0.963
> Q3	0.61 (0.51–0.74)	< 0.001	1.02 (0.82–1.26)	0.855	0.99 (0.78–1.27)	0.981	0.68 (0.61–0.77)	< 0.001	0.98 (0.85–1.13)	0.823	1.04 (0.89–1.22)	0.597
**EPA**												
≤ Q1	Ref		Ref		Ref		Ref		Ref		Ref	
Q1-Q2	1.11 (0.92–1.35)	0.280	0.93 (0.76–1.13)	0.448	0.97 (0.79–1.20)	0.786	1.25 (1.11–1.40)	< 0.001	1.10 (0.98–1.23)	0.112	1.12 (0.98–1.28)	0.094
Q2-Q3	0.89 (0.73–1.10)	0.278	1.01 (0.83–1.21)	0.982	1.05 (0.80–1.38)	0.718	0.85 (0.75–0.96)	0.011	0.96 (0.84–1.10)	0.596	0.98 (0.81–1.19)	0.853
> Q3	0.72 (0.60–0.86)	< 0.001	0.84 (0.72–0.99)	0.033	0.84 (0.63–1.13)	0.244	0.79 (0.70–0.90)	< 0.001	0.92 (0.81–1.04)	0.171	0.95 (0.76–1.18)	0.621
**DPA**												
≤ Q1	Ref		Ref		Ref		Ref		Ref		Ref	
Q1-Q2	0.72 (0.60–0.86)	< 0.001	0.71 (0.60–0.84)	< 0.001	0.72 (0.59–0.87)	< 0.001	0.92 (0.81–1.04)	0.162	0.96 (0.85–1.10)	0.565	1.01 (0.86–1.16)	0.968
Q2-Q3	0.70 (0.58–0.83)	< 0.001	0.83 (0.67–1.02)	0.070	0.79 (0.61–1.03)	0.080	0.78 (0.69–0.89)	< 0.001	0.95 (0.84–1.08)	0.449	1.04 (0.87–1.23)	0.684
> Q3	0.58 (0.49–0.69)	< 0.001	0.85 (0.68–1.05)	0.128	0.81 (0.59–1.11)	0.184	0.68 (0.60–0.77)	< 0.001	0.94 (0.82–1.09)	0.425	1.07 (0.87–1.32)	0.518
**DHA**												
≤ Q1	Ref		Ref		Ref		Ref		Ref		Ref	
Q1-Q2	0.96 (0.79–1.17)	0.689	0.89 (0.75–1.07)	0.218	1.05 (0.87–1.27)	0.621	0.98 (0.85–1.14)	0.823	0.98 (0.85–1.13)	0.792	0.98 (0.83–1.15)	0.791
Q2-Q3	1.03 (0.86–1.24)	0.767	1.04 (0.86–1.25)	0.668	1.26 (0.99–1.61)	0.063	0.92 (0.82–1.04)	0.193	0.94 (0.82–1.08)	0.414	0.95 (0.78–1.15)	0.571
> Q3	0.78 (0.64–0.93)	0.008	0.90 (0.76–1.07)	0.244	1.22 (0.90–1.65)	0.203	0.78 (0.68–0.89)	< 0.001	0.88 (0.77–1.01)	0.069	0.93 (0.73–1.18)	0.534

*PUFA* Polyunsaturated fatty acid, *CVD* Cardiovascular disease, *HR* Hazard ratio, *CI* Confidence interval, *ODA*: Octadecadienoic acid, *Ref* reference, *ALA* Octadecatrienoic acid, *ODTA* Octadecatetraenoic acid, *AA* Eicosatetraenoic acid, *EPA* Eicosapentaenoic acid, *DPA* docosapentaenoic acid, *DHA* Docosahexaenoic acid

Model 1 was the crude model;

Model 2 adjusted for age, gender, race, education level, marital status, PIR, BMI, physical activity, hypertension, DM, smoking, drinking, total energy intake;

Model 3 adjusted for covariates adjusted in Model 2 as well as different PUFAs (excluded the one for analysis)

## Discussion

PUFAs are abundant in many fat-rich foods, especially vegetable oils and fish, and have attracted wide attention due to their important physiological functions in the human body [4, 5]. Our study found that dietary intake of PUFAs such as ODTA, ALA, DPA, and ODA were significantly associated with different CVDs. Higher level of dietary ALA intake was related to lower risks of all-cause mortality, CVD-specific mortality, and other cause-specific mortality. In addition, AA and DPA were also seemed to be benefit to cardiovascular health.

ALA is one of the most common essential PUFAs available in plant sources [23], which has been given much attention to the health benefits of it [12]. A systematic review and meta-analysis basing on 41 prospective cohort studies, containing 1,197,564 participants showed that dietary ALA intake was linked decreased risks of all-causes mortality and CVD-specific mortality [24]. A systematic assessment study also indicated increasing ALA slightly lowered risk of cardiovascular events [25]. Similarly, in the current study, higher dietary ALA intake was associated with both lower risk of all-cause mortality and CVD-specific mortality. It is well known that ALA is a precursor to the long chain n-3 PUFAs, which are critically important for producing various classes of anti-inflammatory eicosanoids [26]. Evidence has suggested ALA might improve CVD risk factors more favorably than other n-3 PUFAs such as EPA and DHA [27]. The role of ALA related to the CVD benefits includes anticoagulant properties, regulating AA-related eicosanoid production, ion flux from cardiac cells, and gene expression, and improving triglyceride and blood pressure [26, 28, 29]. Also, appropriate ALA consumption was seemed to linked to low odds of heart attack. A study on young adults observed that ALA and linoleic acid metabolism pathway metabolites were gradually increased in patients with acute myocardial infarction (AMI), indicating a fatty acid metabolism disorder in AMI in young adults [30]. Further basic research is needed to elucidate the potential mechanism of ALA improvement in heart attacks.

AA is mainly found in the phospholipids of grain-fed animals, dairy products and eggs [31]. Similar to our findings, higher in vivo circulating and tissue levels of AA was linked to lower risk of major cardiovascular events [32]. Wu et al. also indicated that high circulating AA was inversely associated with low risk of CHD-specific mortality in older adults [33]. AA is the predominant long-chain polyunsaturated fatty acids in immune cell membrane phospholipids [34]. Various AA metabolites such as prostaglandins, prostacyclin, and thromboxane A_2_, acting as vasodilators or vasoconstrictors to modulate vascular tone and blood pressure, preventing and managing vascular complications, have antiatherosclerosis effect, and modulate heart function [35–38]. Little has been reported about the role of DPA in CVD-specific mortality. Jiang et al. found a dose–response relationship between an increasing level of DPA and lower risk of CVD [39]. Whereas we did not find DPA to be associated with risk of all-cause mortality, which was consistent with others [37, 38]. Studies have shown that DPA is directly related to the same inflammatory markers as adrenic acid, which may provide a basis for further studies on the metabolism, derivatives, and biological properties of DPA [40].

The main strength of this study is the use of high-quality dietary PUFAs intake data from a well-designed population-based study (NHANES). Also, the associations reported in our study were relatively robust, adjusting for significant confounders established by univariate analysis. However, some limitations were remained in our study. First, despite a comprehensive adjustment for recognized confounders, we could not exclude the possibility of residual or unmeasured confounders. Second, dietary PUFA levels were assessed by 24-h recalls that may result in recalling bias. Third, food intake is susceptible to changes with age and is influenced by a variety of factors [41], although these changes cannot be accounted for in the follow-up period. Larger prospective studies are needed to examine the association of these PUFAs with all-cause mortality as well as CVD-specific mortality. In addition, the NHANES population was Americans, which limited the extrapolation of results to other populations.

## Conclusion

In conclusion, we found that higher dietary ALA intake was associated with decreased risks of all-cause mortality, CVD-specific mortality, and other cause-specific mortality. AA and DPA may also be benefit to cardiovascular health. Given the available evidence, we recommend increasing the intake of PUFAs, such as foods rich in ALA, DPA or AA, to improve the health.

### Supplementary Information

**Additional file 1. **

## Data Availability

The datasets generated and/or analyzed during the current study are available in the NHANES database, https://www.cdc.gov/nchs/nhanes/.

## Abbreviations

CVD: Cardiovascular disease
CHD: Coronary heart disease
CHF: Congestive heart failure
ODA: Octadecadienoic acid
ALA: Alpha-linolenic acid
ODTA: Octadecatetraenoic acid
AA: Eicosatetraenoic acid
EPA: Eicosapentaenoic acid
DPA: Docosapentaenoic acid
DHA: Docosahexaenoic acid
NHANES: National Health and Nutrition Examination Survey
GED: General educational development
SD: Standard deviation
OR: Odds ratio
CI: Confidence interval
HR: Hazard ratio
