# Supplementary material for: Associations of polyunsaturated fatty acids with cardiovascular disease and mortality: a study of NHANES database in 2003–2018
Source: BMC Endocr Disord. 2023 Aug 29;23:185. doi: 10.1186/s12902-023-01412-4 (PMC10464142; doi:10.1186/s12902-023-01412-4)
Supplement: Supplementary file 1 — Additional file 1. [file 12902_2023_1412_MOESM1_ESM.docx]

**Table S1. The quartile of the PUFAs intake**

| Dietary PUFAs | Q1 | Q2 | Q3 |
| --- | --- | --- | --- |
| Octadecadienoic acid (ODA), gm | 8.929667 | 14.12298 | 21.141 |
| Octadecatrienoic acid (ALA), gm | 0.853 | 1.379976 | 2.129 |
| Octadecatetraenoic acid (ODTA), gm | 0 | 0 | 0.002815 |
| Eicosatetraenoic acid (AA), gm | 0.057 | 0.112 | 0.201 |
| Eicosapentaenoic acid (EPA), gm | 0.001499 | 0.006012 | 0.015169 |
| Docosapentaenoic acid (DPA), gm | 0.00235 | 0.012 | 0.026 |
| Docosahexaenoic acid (DHA), gm | 0.001851 | 0.014612 | 0.05877 |
| Total PUFAs, gm | 10.256 | 16.04637 | 23.83346 |

PUFA: polyunsaturated fatty acid, Q1: 1st quartile, Q2: 2nd quartile, Q3: 3rd quartile
